# Evaluation of Suppressiveness of Soils Exhibiting Soil-Borne Disease Suppression after Long-Term Application of Organic Amendments by the Co-cultivation Method of Pathogenic *Fusarium oxysporum* and Indigenous Soil Microorganisms

**DOI:** 10.1264/jsme2.ME17072

**Published:** 2018-03-29

**Authors:** Masahiro Mitsuboshi, Yuuzou Kioka, Katsunori Noguchi, Susumu Asakawa

**Affiliations:** 1 Tsukuba Research Institute, Katakura & Co-op Agri Corporation 5–5511 Namiki, Tsuchiura, Ibaraki 300–0061 Japan; 2 Katakura & Co-op Agri Corporation 1–8–10 Kudankita, Chiyoda, Tokyo 102–0073 Japan; 3 Graduate School of Bioagricultural Sciences, Nagoya University 1 Furo-cho, Chikusa, Nagoya, Aichi 464–8601 Japan

**Keywords:** soil-borne disease suppression, long-term application of organic amendments, biological diagnosis, *Fusarium oxysporum*, disease incidence

## Abstract

Preventive measures against soil-borne diseases need to be implemented before cultivation because very few countermeasures are available after the development of diseases. Some soils suppress soil-borne diseases despite the presence of a high population density of pathogens. If the suppressiveness of soil against soil-borne diseases may be predicted and diagnosed for crop fields, it may be possible to reduce the labor and cost associated with excessive disinfection practices. We herein evaluated the suppressiveness of soils in fields with the long-term application of organic amendments by examining the growth of pathogenic *Fusarium oxysporum* co-cultivated with indigenous soil microorganisms on agar plates. Soils treated with coffee residue compost or rapeseed meal showed suppressiveness against spinach wilt disease by *F. oxysporum* f. sp. *spinaciae* or spinach wilt and lettuce root rot diseases by *F. oxysporum* f. sp. *spinaciae* and *F. oxysporum* f. sp. *lactucae*, respectively, and the growth of pathogenic *Fusarium* spp. on agar plates was suppressed when co-cultured with microorganisms in a suspension from these soils before crop cultivation. These results indicate the potential of the growth degree of pathogenic *F. oxysporum* estimated by this method as a diagnostic indicator of the suppressiveness of soil associated with the inhabiting microorganisms. A correlation was found between the incidence of spinach wilt disease in spinach and the growth degree of *F. oxysporum* f. sp. *spinaciae* by this co-cultivation method, indicating that suppressiveness induced by organic amendment applications against *F. oxysporum* f. sp. *spinaciae* is evaluable by this method. The co-cultivation method may be useful for predicting and diagnosing suppressiveness against soil-borne diseases.

Plant diseases include those on the above-ground part of plants and soil-borne diseases. Effective countermeasures against above-ground diseases are possible by diagnosing the initial incidence of disease. Although this is not the case for soil-borne diseases, resistant cultivars, cultural pest control, and biological pesticides may be employed as countermeasures against these diseases (11). The pathogenic fungi causing soil-borne diseases spread throughout a field, and it is often too late to employ countermeasures when the early symptoms of a disease are detected. Thus, preventive measures need to be implemented before cultivation.

Some soils suppress soil-borne diseases despite the presence of a high population density of pathogens and are known as “suppressive soil”. The physicochemical and biological characteristics of soil contribute to this suppressiveness (5). The effects of organic amendments, such as livestock and green manure, organic waste, compost, and peat, on the suppression of soil-borne pathogens have been extensively examined (6). If the suppressiveness of soil induced by various management approaches such as the application of organic fertilizers may be predicted and diagnosed for crop fields, it will be possible to reduce the labor and cost associated with excessive disinfection practices.

Pathogenic *F. oxysporum* causes serious damage to various crop species, and the control of diseases caused by *F. oxysporum* is very challenging. Bonanomi *et al.* (6) analyzed an extensive data set of 2,423 studies to identify the characteristics of organic amendments to soil related to suppressiveness against soil-borne diseases. They found that the most useful features showing positive correlations with the disease suppression of *Fusarium* spp. were total fungi, total bacteria, the C to N ratio, pH, fluorescent pseudomonads, EC, and sporegenous bacteria. Previous studies investigated suppressiveness against *Fusarium* diseases. Very diverse factors including biotic and abiotic factors are involved in the expression of the suppressiveness of soil against *Fusarium* diseases. For example, the suppression of *F. oxysporum* f. sp. *raphani* by *Pseudomonas stutzeri* attaching to chlamydospores (14), the antagonism to *F. oxysporum* f. sp. *raphani* by microorganisms colonizing radish roots (15), the suppression of *F. oxysporum* f. sp. *spinaciae* by increases in microbial activity and populations after the application of compost (8), and the suppression of *F. oxysporum* f. sp. *cubense* by toxic organic acids produced in biological soil disinfestation (10) have been reported. It is not possible to examine all of these factors for their soil suppressiveness against *Fusarium* diseases, and those related to suppressiveness may vary depending on the crop, *Fusarium* species, and soil conditions. Therefore, the suppressiveness of soil against *Fusarium* diseases needs to be comprehensively assessed.

In order to evaluate soil suppressiveness against soil-borne diseases, inoculation experiments of pathogens to plants are required under crop growing conditions. However, these experiments are laborious and a long time is needed to observe the occurrence of diseases. It is practically impossible to conduct these experiments as a preventive diagnosis in farmers’ fields because the implementation of countermeasures against soil-borne diseases needs to occur before crop planting. Therefore, a new method that evaluates suppressiveness against soil-borne diseases in a short time is required. We herein employed and investigated the ability of a simple method to evaluate the suppressiveness of soil microorganisms against pathogenic *F. oxysporum* f. sp. *spinaciae* by co-cultivating the pathogenic fungus with indigenous soil microorganisms using the dilution plate technique (12). This method (the co-cultivation method) comprehensively estimates suppressiveness against a pathogen by the abundance, activity, and antagonistic ability of indigenous soil microorganisms. Suppressiveness against *F. oxysporum* f. sp. *spinaciae* was found in cow dung compost and a microbial inoculant, and the incidence of disease in spinach by the pathogen was reduced in soils treated with these organic amendments. Furthermore, positive correlations were observed between the incidence of disease in spinach and the growth degree of *F. oxysporum* f. sp. *spinaciae* on agar plates in an inoculation experiment of the pathogen to spinach in soils treated with several types of organic amendments (12). However, since these findings were obtained from a pot scale experiment using one type of soil, the applicability of the co-cultivation method to field soil with crop cultivation needs to be confirmed.

In the present study, the co-cultivation method (12) for evaluating soil suppressiveness against pathogenic *Fusarium* spp. was examined using field soils with the long-term application of organic amendments that exhibited suppressiveness. Inoculation experiments of pathogenic *F. oxysporum* f. sp. *spinaciae* (spinach wilt disease) and *F. oxysporum* f. sp. *lactucae* (lettuce root rot disease) to spinach and Boston lettuce, respectively, were performed for soils from two long-term experimental fields, and relationships between the disease incidence of plants and the suppressive degrees of growth of *Fusarium* spp. estimated by the co-cultivation method for soil before and after crop cultivation were investigated.

## Materials and Methods

### Soil

Soil samples were taken from a long-term experimental field with the application of organic fertilizers from two locations: Aichi prefecture and Ibaraki prefecture, Japan. Soil samples in Aichi prefecture (Aichi soil) were collected at the Nagoya University Farm, Graduate School of Bioagricultural Sciences, Togo-cho, Aichi, Japan, on March 8, 2016. This field experiment has been continued since 1987 and soil was Yellow soil (Ultisols). Five plots were used: unfertilized plot (NF), a plot with chemical fertilizer (CF, 500 kg N ha^−1^ y^−1^, 133 kg P_2_O_5_ ha^−1^ y^−1^, 500 kg K_2_O ha^−1^ y^−1^), a plot with chemical fertilizer and 40 t ha^−1^ y^−1^ of farmyard manure (CF+FYM), a plot with chemical fertilizer and 40 t ha^−1^ y^−1^ of coffee residue compost (CRC), and a plot with 400 t ha^−1^ y^−1^ of farmyard manure before 2006 or 200 t ha^−1^ y^−1^ of farmyard manure after 2006 (FYM). Each plot was 100 m^2^ (4×25 m) without replication. The cultivation history of this field is shown in Supporting information.

Soil samples in Ibaraki prefecture (Ibaraki soil) were collected at the Tsukuba Research Institute Farm, Katakura Chikkarin, Tsuchiura, Ibaraki, Japan on March 24, 2015. This field experiment has been continued since 1987 and the soil was “Kuroboku” soil (Andosol). Five plots were used: a plot with inorganic fertilizers (ammonium sulfate, calcium superphosphate, and potassium chloride) (Cont), a plot with rapeseed meal (RSM, N-P_2_O_5_-K_2_O=5.3-2-1%, 940–4,700 kg ha^−1^), a plot with fish meal (FM, N-P_2_O_5_-K_2_O=7-7-0%, 710–3,600 kg ha^−1^), a plot with steamed bone meal (SBM, N-P_2_O_5_-K_2_O=4-20-0%, 1,300–6,300 kg ha^−1^), and a plot with a mixture of these organic fertilizers (Mix, N-P_2_O_5_-K_2_O=5.4-9.6-0.3%, 930–4,600 kg ha^−1^). Each plot was 10 m^2^ in triplicate. The cultivation history of this field is shown in Supporting information.

The soil samples used for the inoculation experiment were placed in a plastic bag or container with a lid to avoid drying and stored at room temperature.

### Chemical and microbial characteristics of soil

The pH and electrical conductivity (EC) of soil were measured in a water extract (5:1 [v/v]) with the pH meter M-12 (Horiba, Tokyo, Japan) and EC meter ES-51 (Horiba). The concentrations of elements were measured using the Soil & Plant Analyzer SFP-4i (Fujihira Industry, Tokyo, Japan) following the manufacturer’s instructions for soil samples air-dried at room temperature and passed through a 2-mm sieve.

The population density of fungi was assessed by the dilution plate technique on rose bengal agar medium (KH_2_PO_4_ 1 g, MgSO_4_·7H_2_O 0.5 g, peptone 5 g, glucose 10 g, rose bengal 33 mg, agar 17 g, streptomycin 30 mg, and distilled water 1 L; pH 6.8) (3). The populations of actinomycetes and bacteria were evaluated by the dilution plate technique on egg albumin agar medium (egg albumin 0.25 g, glucose 1 g, K_2_HPO_4_ 0.5 g, MgSO_4_·7H_2_O 0.2 g, Fe_2_ [SO_4_]_3_ trace, agar 15 g, and distilled water 1 L; pH 6.8) (3).

The diversity of bacterial communities was investigated by analyzing the 16S rRNA sequence of Ibaraki soil. DNA was extracted from 0.5 g of soil using the FastDNA SPIN kit for Soil (MP Biomedicals, Santa Ana, CA, USA) and sequencing was performed with a MiSeq (Illumina, San Diego, CA, USA). In the analysis of alpha diversity, data of an array were sampled in accordance with the minimum number of reads with a threshold value of 97% using QIIME’s workflow script. Measurements were conducted for triplicate plots, except for Mix in which one of the triplicate plots was used.

### Pathogenic fungal strain

Spinach wilt disease fungus (*F. oxysporum* f. sp. *spinaciae* MAFF 103060) and lettuce root rot fungus (*F. oxysporum* f. sp. *lactucae* MAFF 744028) were used.

### Crop

Spinach (*Spinacia oleracea* L.) (“OKAME”, TAKII, Kyoto, Japan) and Boston lettuce (*Lactuca sativa* L. var. *capiata*) (“OKAYAMA SARADANA”, TAKII) were used.

### Cultivation of crops and inoculation of the pathogenic *F. oxysporum* strain

Spinach seeds pretreated in water for 2 d were sown into a cell tray (200 holes) filled with nursery soil (“YASAI-BAIDO ICHI GOU”, Katakura & Co-op Agri, Tokyo, Japan) and grown in a greenhouse for approximately 10 d. Conidiospores of *F. oxysporum* f. sp. *spinaciae* were added to the soil sample collected from the long-term experimental field at a dose of 10^6^ conidia g^−1^ soil, and 500 mL of soil (approximately 400 g) was placed into polycarbonate pots (outer diameter of 12 cm × height of 11.5 cm; 0.01 m^2^). Regarding the proliferation of conidiospores, *F. oxysporum* was cultivated on potato dextrose agar (potato extract [prepared from 1 kg potatoes boiled in 1 L of water] 100 mL, glucose 20 g, agar 15 g, and distilled water 900 mL) at 30°C for 7 d, and a square section of 5 mm on each side of the fungal lawn was cultivated in 100 mL of potato sucrose broth (potato extract 100 mL, sucrose 20 g, and distilled water 900 mL) at 30°C for 7 d by shaking horizontally. The number of conidiospores was enumerated on a hemocytometer and diluted culture solutions with a predetermined density were used for the inoculation. Soils were fertilized with the compound inorganic fertilizer (N-P-K=150-300-150 kg ha^−1^), inoculated with the conidiospores, and planted with the spinach seedlings on the same day. Three spinach seedlings were planted in each pot in quadruplicate on April 22, 2016 (Aichi soil) and September 24, 2015 (Ibaraki soil). We noted the disease incidence and severity of wilting for each spinach plant on May 7, 2016 (15 d after planting, Aichi soil) and October 6, 2015 (12 d after planting, Ibaraki soil) and collected soil samples from the pots in order to measure the growth degree of *F. oxysporum*. The incidence and severity of wilt were evaluated as follows: 0, healthy; 1, one leaf had wilted; 2, two or three leaves had wilted; 3, half of the leaves had wilted; 4, more than half of the leaves had wilted; 5, dead or nearly dead.

Boston lettuce seeds were sown into a cell tray (200 holes) filled with nursery soil (“YASAI-BAIDO ICHI GOU”) and grown on a cultivation shelf in the laboratory, which was kept at 25°C. Conidiospores of *F. oxysporum* f. sp. *lactucae* were added to the soils at a dose of 10^6^ conidia g^−1^ soil and 500 mL of the soil (approximately 400 g) was placed into polycarbonate pots (outer diameter of 12 cm × height of 11.5 cm; 0.01 m^2^). Soils were fertilized with the compound inorganic fertilizer (N-P-K=150-300-150 kg ha^−1^), inoculated with the conidiospores, and planted with the Boston lettuce seedlings on the same day. Three Boston lettuce seedlings were planted in each pot in triplicate on July 26, 2016 for both soils. We recorded the disease incidence and severity of wilting for each Boston lettuce plant on August 8, 2016 (13 d after planting) and collected soil samples from the pots in order to measure the growth degree of *F. oxysporum*. The incidence and severity of wilt were evaluated in the same manner as that described above for spinach.

The cultivation of spinach and Boston lettuce inoculated with the respective pathogenic fungus was also performed using sterilized CRC, FYM, and RSM soils. Soil was autoclaved at 121°C for 60 min in a bag and the inoculation test was performed in the same manner as the above method.

Soil samples were subjected to analyses by the co-cultivation method within two weeks of sampling and stored at 4°C before being analyzed.

### Co-cultivation of *F. oxysporum* with soil microorganisms

Ten-gram portions of the soil sample, which was collected in triplicate before crop cultivation and from pots in quadruplicate for spinach or triplicate for Boston lettuce for each treatment after crop cultivation, were taken into a sterilized tube containing 90 mL of sterilized tap water and shaken reciprocally at 200 rpm for 30 min. One milliliter of the suspension was poured into 9 mL of sterilized tap water, mixed well, and serially diluted in the same manner. A dilution series was prepared to a magnification of 10^−6^ fold. A quantity of 1.0 mL of 10^−1^ to 10^−6^-fold diluted suspensions was inoculated into a petri dish and 16 mL of YPMG agar medium (Peptone-yeast extract medium; yeast extract 3 g, peptone 5 g, beef extract 3 g, glucose 10 g, agar 15 g, and distilled water 1 L; pH 7.0) (2) was poured and mixed. A square section of the *F. oxysporum* hyphal lawn was placed in the center of agar medium. As a control, sterilized water was inoculated instead of the diluted suspension of soil samples. Plates were incubated at 30°C for approximately one week (7 or 8 d), by which time the colony of *F. oxysporum* had spread fully on the control plate. The length of the shortest part of the colony together with the longest length was measured; *i.e.*, the extension of hyphae was measured at the parts at which hyphae had grown the most (long diameter) and least (short diameter) in the colony of *F. oxysporum* for soil samples and the control, and the mean of these values was used to calculate the growth degree. As a representative value for the growth degree of *F. oxysporum*, the median of the estimated values of the growth degree at six dilutions from 10^−1^ to 10^−6^ was calculated (12).

### Statistical analysis

All statistical tests were performed with Microsoft Excel 2016 for Windows (Microsoft, Tokyo, Japan) and Ekuseru-Toukei 2015 (Social Survey Research Information, Tokyo, Japan). A principal component analysis was performed using the data of pH, EC, NH_3_-N, exchangeable Mg, bacterial density, and fungal density. These parameters were selected by assessing similarities using a cluster analysis (Ward’s method) assuming the distance between variables using the correlation coefficient between variables. Differences in the disease incidence of spinach and Boston lettuce and the growth degree of *F. oxysporum* in the co-cultivation method from those in control plots (NF and Cont) were statistically tested with Steel’s and Dunnett’s tests, respectively. The relationship between the disease incidence of crops and the growth degree of *F. oxysporum* estimated by the co-cultivation method was analyzed by Spearman’s rho.

## Results

### Chemical and microbial characteristics of soil

The chemical and microbial characteristics of Aichi and Ibaraki soil samples are shown in Table S1. Several characteristics were significantly different between Cont and the other plots with organic amendments in Ibaraki soil. PCA was performed based on chemical and microbial characteristics (Fig. S1). Cont, CF, and NF were located on the second and third quadrants and the plots with organic amendments were located from the center to the first and fourth quadrants. FYM and CRC were located apart from the other plots with organic amendments in the fourth and first quadrants, respectively. The diversity of bacterial communities in Ibaraki soil based on the elucidation of 16S rRNA sequences showed no significant difference among plots (Table S2).

### Disease incidence of spinach and Boston lettuce planted in soil with the long-term application of organic amendments

**Fig. 1:**
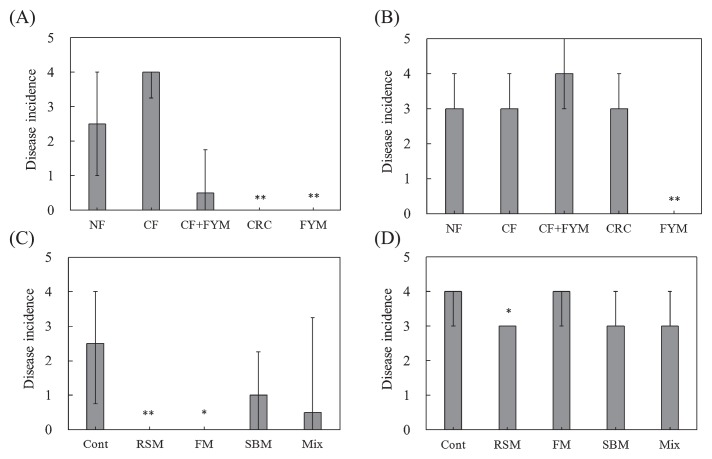
Disease incidence of spinach by *Fusarium oxysporum* f. sp. *spinaciae* (A, C) and Boston lettuce by *F. oxysporum* f. sp. *lactucae* (B, D) in Aichi soil (A, B) and Ibaraki soil (C, D). NF, unfertilized; CF, chemical fertilizer; CF+FYM, chemical fertilizer and 40 t ha^−1^ y^−1^ farmyard manure; CRC, chemical fertilizer and 40 t ha^−1^ y^−1^ coffee residue compost; FYM, 400 t ha^−1^ y^−1^ farmyard manure. Cont, compound inorganic fertilizers; RSM, 940–4,700 kg ha^−1^ rapeseed meal; FM, 710–3,600 kg ha^−1^ fish meal; SBM, 1,300–6,300 kg ha^−1^ steamed bone meal; Mix, 930–4,600 kg ha^−1^ mixture of rapeseed meal, fish meal, and steamed bone meal. Values show the median with the upper and lower quartile points (A, C: *n*=12; B, D: *n*=9). * and ** indicate significant differences from NF or Cont at *P*<0.05 and *P*<0.01, respectively.

In NF and Cont, a large number of leaves wilted, while disease incidence was relatively suppressed in CRC, FYM, and RSM (Fig. S2). The disease incidence of spinach was significantly lower in CRC (*P*=0.001) and FYM (*P*=0.000) than in NF in Aichi soil, and was also significantly lower in RSM (*P*=0.007) and FM (*P*=0.023) than in Cont in Ibaraki soil (Fig. 1A and C). The disease incidence of Boston lettuce was significantly lower in FYM (*P*=0.001) and RSM (*P*=0.042) than in NF and Cont in Aichi and Ibaraki soils, respectively (Fig. 1B and D). When soil was sterilized, the disease incidence of spinach increased in CRC, whereas it was maintained at a low level in FYM and RSM. The disease incidence of Boston lettuce increased in CRC and FYM, but did not significantly increase in RSM after soil sterilization (Fig. S3). Disease was observed in spinach or Boston lettuce grown on soil without the inoculation of pathogenic *F. oxysporum* (data not shown).

### Growth degree of *F. oxysporum* for soil

**Fig. 2:**
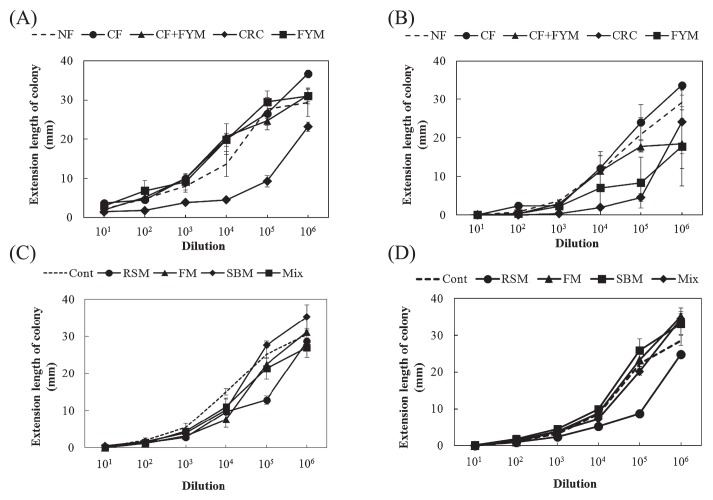
Growth degree of *Fusarium oxysporum* f. sp. *spinaciae* based on an estimation of the extension length of the colony for Aichi (A, B) and Ibaraki (C, D) soils with the cultivation of spinach. Values show the mean of growth degrees at each dilution with SE before (A, C) (*n*=3) and after crop cultivation (B, D) (*n*=4). NF, unfertilized; CF, chemical fertilizer; CF+FYM, chemical fertilizer and 40 t ha^−1^ y^−1^ farmyard manure; CRC, chemical fertilizer and 40 t ha^−1^ y^−1^ coffee residue compost; FYM, 400 t ha^−1^ y^−1^ farmyard manure; Cont, compound inorganic fertilizers; RSM, 940–4,700 kg ha^−1^ rapeseed meal; FM, 710–3,600 kg ha^−1^ fish meal; SBM, 1,300–6,300 kg ha^−1^ steamed bone meal; Mix, mixture of rapeseed meal, fish meal, and steamed bone meal. The extension lengths for control plates were 42.0 (A), 37.0 (B), 40.0 (C), and 39.0 (D) mm.

The growth degree of *F. oxysporum* f. sp. *spinaciae* was significantly lower in CRC (*P*=0.000) than in NF before spinach cultivation in Aichi soil (Fig. 2A). After crop cultivation, the growth degree of *F. oxysporum* was significantly lower in CRC (*P*=0.011) and FYM (*P*=0.029) than in NF (Fig. 2B). In Ibaraki soil, the degree of *F. oxysporum* proliferation was significantly lower in RSM than in Cont (*P*=0.001 [before crop cultivation] and *P*=0.000 [after crop cultivation], Fig. 2C and D).

**Fig. 3:**
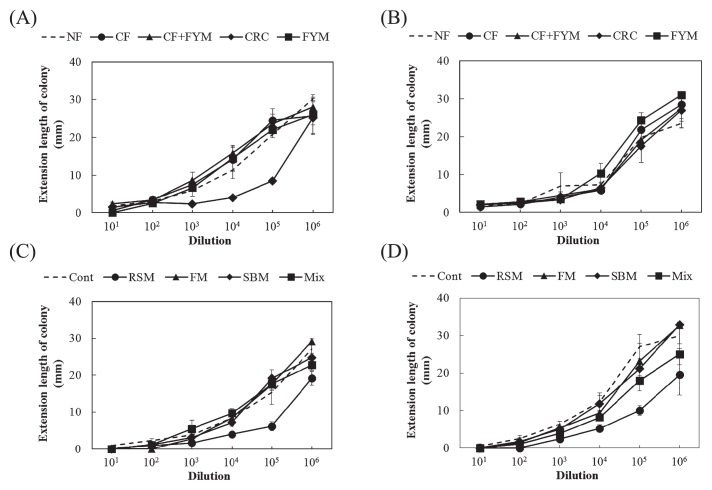
Growth degree of *Fusarium oxysporum* f. sp. *lactucae* based on an estimation of the extension length of the colony for Aichi (A, B) and Ibaraki (C, D) soils with the cultivation of Boston lettuce. Values show the mean of growth degrees at each dilution with SE before (A, C) (*n*=3) and after crop cultivation (B, D) (*n*=3). NF, unfertilized; CF, chemical fertilizer; CF+FYM, chemical fertilizer and 40 t ha^−1^ y^−1^ farmyard manure; CRC, chemical fertilizer and 40 t ha^−1^ y^−1^ coffee residue compost; FYM, 400 t ha^−1^ y^−1^ farmyard manure; Cont, compound inorganic fertilizers; RSM, 940–4,700 kg ha^−1^ rapeseed meal; FM, 710–3,600 kg ha^−1^ fish meal; SBM, 1,300–6,300 kg ha^−1^ steamed bone meal; Mix, 930–4,600 kg ha^−1^ mixture of rapeseed meal, fish meal, and steamed bone meal. The extension lengths for control plates were 36.0 (A, B) and 35.0 (C, D) mm.

The growth degree of *F. oxysporum* f. sp. *lactucae* was significantly lower in CRC (*P*=0.001) than in NF before Boston lettuce cultivation in Aichi soil (Fig. 3A). After crop cultivation, no significant differences were observed in the growth degree of *F. oxysporum* (Fig. 3B). Before crop cultivation in Ibaraki soil, the growth degree of *F. oxysporum* was significantly lower in RSM (*P*=0.000) than in Cont (Fig. 3C). After crop cultivation, the growth degree of *F. oxysporum* was significantly lower in RSM (*P*=0.000) and Mix (*P*=0.005) than in Cont (Fig. 3D).

**Fig. 4:**
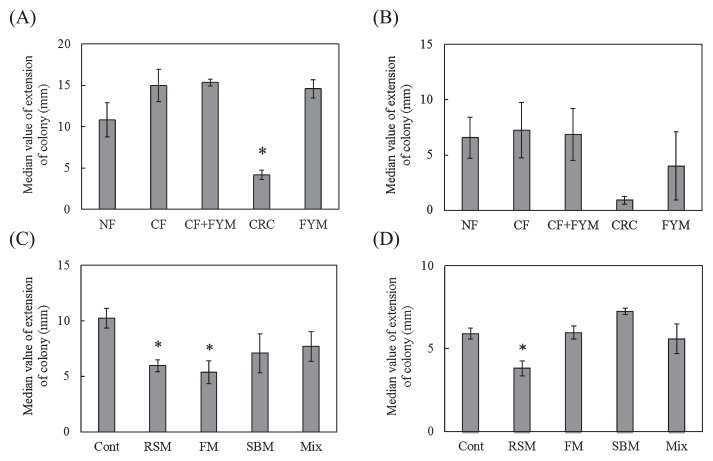
Growth degree of *Fusarium oxysporum* f. sp. *spinaciae* based on an estimation of the extension length of the colony for the cultivation of spinach in Aichi (A, B) and Ibaraki (C, D) soils. Values show the mean of medians at dilutions from 10^−1^ to 10^−6^ with SE before (A, C) (*n*=3) and after crop cultivation (B, D) (*n*=4). NF, unfertilized; CF, chemical fertilizer; CF+FYM, chemical fertilizer and 40 t ha^−1^ y^−1^ farmyard manure; CRC, chemical fertilizer and 40 t ha^−1^ y^−1^ coffee residue compost; FYM, 400 t ha^−1^ y^−1^ farmyard manure; Cont, compound inorganic fertilizers; RSM, 940–4,700 kg ha^−1^ rapeseed meal; FM, 710–3,600 kg ha^−1^ fish meal; SBM, 1,300–6,300 kg ha^−1^ steamed bone meal; Mix, 930–4,600 kg ha^−1^ mixture of rapeseed meal, fish meal, and steamed bone meal. * indicates a significant difference from NF or Cont at *P*<0.05.

The representative value of the growth degree based on the extension length of the colonies (median value) was significantly lower for CRC than for NF (*P*=0.023) before spinach cultivation in Aichi soil (Fig. 4A). After crop cultivation, the median value of *F. oxysporum* was not significantly different in Aichi soil (Fig. 4B). Before crop cultivation in Ibaraki soil, median values were significantly lower in RSM (*P*=0.041) and FM (*P*=0.023) than in Cont (Fig. 4C). The median value was significantly lower in RSM (*P*=0.035) than in Cont (Fig. 4D) after crop cultivation.

**Fig. 5:**
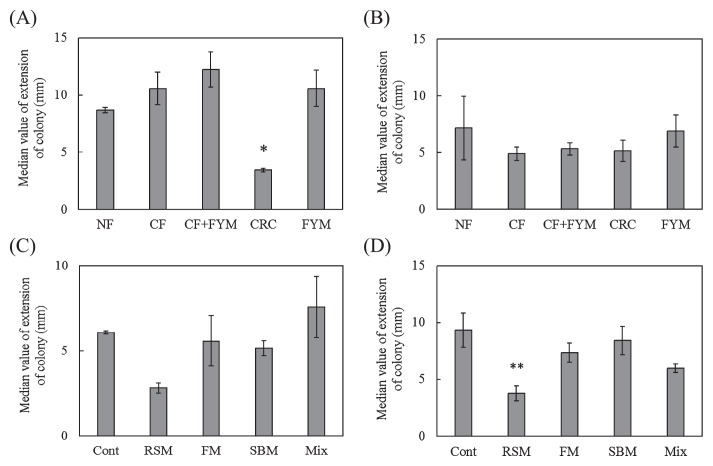
Growth degree of *Fusarium oxysporum* f. sp. *lactucae* based on an estimation of the extension length of the colony for the cultivation of Boston lettuce in Aichi (A, B) and Ibaraki (C, D) soils. Values show the mean of medians at dilutions from 10^−1^ to 10^−6^ with SE before (A, C) (*n*=3) and after crop cultivation (B, D) (*n*=3). NF, unfertilized; CF, chemical fertilizer; CF+FYM, chemical fertilizer and 40 t ha^−1^ y^−1^ farmyard manure; CRC, chemical fertilizers and 40 t ha^−1^ y^−1^ coffee residue compost; FYM, 400 t ha^−1^ y^−1^ farmyard manure; Cont, compound inorganic fertilizers; RSM, 940–4,700 kg ha^−1^ rapeseed meal; FM, 710–3,600 kg ha^−1^ fish meal; SBM, 1,300–6,300 kg ha^−1^ steamed bone meal; Mix, 930–4,600 kg ha^−1^ mixture of rapeseed meal, fish meal, and steamed bone meal. * and ** indicate significant differences from NF or Cont at *P*<0.05 and *P*<0.01, respectively.

The median value of *F. oxysporum* f. sp. *lactucae* was significantly lower in CRC (*P*=0.033) than in NF before Boston lettuce cultivation in Aichi soil (Fig. 5A). The median value of *F. oxysporum* f. sp. *lactucae* was not significantly different after crop cultivation in Aichi soil or before crop cultivation in Ibaraki soil (Fig. 5B and C). The median value was significantly lower in RSM (*P*=0.011) than in Cont after Boston lettuce cultivation in Ibaraki soil (Fig. 5D).

### Relationship between the growth degrees of *F. oxysporum* and disease incidence of plants

**Fig. 6:**
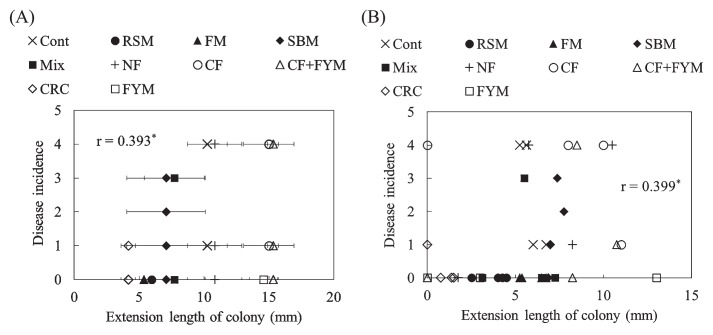
Correlation between the disease incidence of spinach by *Fusarium oxysporum* f. sp. *spinaciae* in each plant and the mean of median values of growth degrees of *F. oxysporum* f. sp. *spinaciae* with SE (A) on an estimation of the extension length of the colony (*n*=40) before (A) and after crop cultivation (B). * indicates a significant difference at *P*<0.05.

**Fig. 7:**
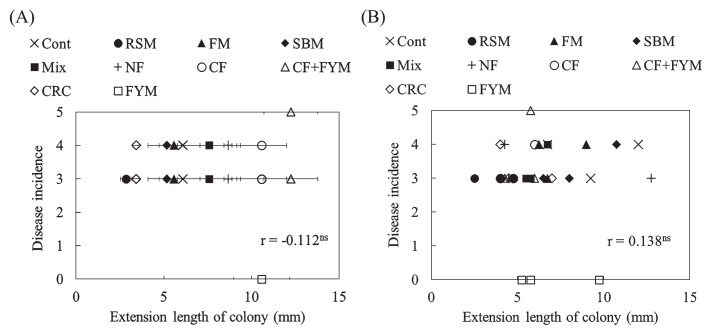
Correlation between the disease incidence of Boston lettuce by *Fusarium oxysporum* f. sp. *lactucae* in each plant and the mean of median values of growth degrees of *F. oxysporum* f. sp. *lactucae* with SE (A) on an estimation of the extension length of the colony (*n*=30) before (A) and after crop cultivation (B).

The relationship between the disease incidence of plants and growth degrees of *F. oxysporum* in Aichi and Ibaraki soils is shown in Fig. 6 (spinach, *F. oxysporum* f. sp. *spinaciae*) and 7 (Boston lettuce, *F. oxysporum* f. sp. *lactucae*), respectively. Positive correlations were found between the disease incidence of spinach and growth degree of *F. oxysporum* f. sp. *spinaciae* based on the extension length of the colonies before (*P*=0.012) and after crop cultivation (*P*=0.011) (Fig. 6). A correlation was not observed between the disease incidence of Boston lettuce and growth degree of *F. oxysporum* f. sp. *lactucae* (*P*=0.556 [before crop cultivation] and *P*=0.467 [after crop cultivation], Fig. 7).

## Discussion

Decreases in the disease incidence of plants indicated that CRC, FYM, RSM, and FM soils expressed suppressiveness against spinach wilt disease, while FYM and RSM soils expressed suppressiveness against lettuce root rot disease (Fig. 1 and S2). Since suppressiveness against soil-borne diseases may be developed by the application of organic amendments (6), the long-term application of organic amendments in fields may have contributed to the suppressiveness of soils. Before crop cultivation, the growth of *F. oxysporum* f. sp. *spinaciae* and *F. oxysporum* f. sp. *lactucae* was suppressed more in CRC than in NF as well as in RSM than in Cont (Fig. 2 and 3). These results suggest that the co-cultivation method has the capacity to evaluate suppressiveness against soil-borne diseases of soils before crop cultivation in fields with the long-term application of organic amendments.

A positive correlation was observed between the disease incidence of spinach and growth degree of *F. oxysporum* f. sp. *spinaciae* (Fig. 6), as reported in the pot experiment (12). When the co-cultivation method is used to diagnose soil suppressiveness against *Fusarium* diseases, it will be necessary to reveal the relationship between the growth suppression degree of pathogenic *Fusarium* strains by the co-cultivation method and the disease incidence of crops. The disease incidence of spinach was low when the growth degree was less than approximately 5 mm (Fig. 6), indicating that a growth degree of 5 mm may be an index for a diagnosis. However, caution is needed regarding an outlier observed in CF (Fig. 6B); despite the high incidence of 4, the growth degree of *F. oxysporum* f. sp. *spinaciae* was 0 mm. On the 10^4^-fold dilution plate, the colony of *F. oxysporum* was covered with the lawn of a fungus and the growth of *F. oxysporum* was inhibited, resulting in an extension length of the colony of 0 mm. Thus, care is needed in this case because the growth degree of *F. oxysporum* estimated by the co-cultivation method may be affected and, thus, does not correspond to the disease incidence of plants. Further investigations of the relationship between the growth degree of pathogenic *Fusarium* strains estimated by the co-cultivation method and the disease incidence of plants are needed before the application of the co-cultivation method for practical use.

A correlation was not observed between the disease incidence of Boston lettuce and growth degree of *F. oxysporum* f. sp. *lactucae* (Fig. 7), indicating that the co-cultivation method is not useful for evaluating soil suppressiveness against lettuce root rot disease. However, RSM showed the growth suppression of *F. oxysporum* f. sp. *lactucae* by the co-cultivation method and disease incidence was reduced in RSM (Fig. 1D, 3D, and 5D). The disease incidence of Boston lettuce was mostly 3 or 4 and this may have affected the evaluation of the growth degree by the method and resulted in no relationship between the disease incidence and degree of *F. oxysporum* f. sp. *lactucae*. The difference observed in the degree of suppressiveness between spinach and Boston lettuce observed in CRC (Fig. 1) may have indicated that the fungistatic capability of these soils was less effective for *F. oxysporum* f. sp. *lactucae* than for *F. oxysporum* f. sp. *spinaciae*. There are very diverse pathogenic types (Formae speciales) of *F. oxysporum* (4) and differences in their infectivities may have contributed to this difference. In addition, lettuce root rot disease showed symptoms with a low density of *F. oxysporum* f. sp. *lactucae* in soil (13). Therefore, it was not possible to evaluate suppressiveness using the co-cultivation method; however, the growth degree of *Fusarium* was assessed using this method.

Disease suppression for spinach wilt was not shown in the sterilized soil of CRC, indicating that biotic factors were involved in the suppressiveness of CRC. The population density of fungi in CRC was two orders of magnitude higher than that in other plots and the fungal community structure may be one of the factors related to the suppressiveness of CRC for spinach wilt disease; however, suppressiveness was not observed for lettuce root rot disease. Adams *et al.* (1) reported that spent coffee grounds reduced the population density of *F. solani* f. sp. *phaseoli*. Hamanaka *et al.* (9) also showed suppressiveness against crown and root rot of tomato caused by *F. oxysporum* f. sp. *radices-lycopersici* in CRC soil. They demonstrated that fungi were predominant in the microbial community and suggested that fungal members close to *F. oxysporum* were responsible for this suppressiveness. Fungi related to *Fusarium* in soil may also have been involved in the suppression of *F. oxysporum* f. sp. *spinaciae* on plates of up to 10^−4^-fold dilutions because the population density of *Fusarium* spp. was estimated to be approximately 10^4^ cfu g^−1^ in CRC soil (data not shown).

Suppressiveness against spinach wilt disease was observed in the sterilized soil of RSM, indicating that abiotic factors are involved in the suppressiveness of RSM. Ueda *et al.* (16) reported that composted rapeseed meal exhibited lytic activity against *F. oxysporum* f. sp. *cucumerium* and reduced the population density of *F. oxysporum* in soil. Zakaria *et al.* (18) found that volatile inhibitory substances including ammonia were involved in the reduction of *F. oxysporum* and *F. solani* in soils treated with oilseed (linseed, cottonseed, and soybean) meals. Some of the substances involved in suppressiveness produced during the decomposition process of rapeseed meal may have been heat-stable, whereas substances such as ammonia were degraded or removed from soil by autoclaving. In addition, suppression of the growth of *F. oxysporum* was observed using the *Fusarium* co-cultivation method in RSM (Fig. 4C and D), indicating that biotic factors were also involved in suppressiveness in RSM. Members of *Actinobacteria* were previously suggested to play a key role as microbial defenders in suppressive soil against *Fusarium* wilt of strawberry caused by *F. oxysporum* f. sp. *fragariae* (7). The population density of actinomycetes was higher in RSM than in Cont (Table 1). We observed higher number of colonies of actinomycetes on the 10^−5^-fold dilution plates for RSM than on the plates for other plots, indicating that the suppression of *Fusarium* is related to actinomycetes.

Although FYM exhibited suppressiveness against spinach wilt and lettuce root rot diseases (Fig. 1A and B), the co-cultivation method showed no effect on the suppression of the growth of *F. oxysporum* f. sp. *spinaciae* (Fig. 2A and 4A). Abiotic factors may have been involved in this suppressiveness because the sterilized soil of FYM still showed suppressiveness in spinach. Ueda *et al.* (17) reported that sterols extracted from bark compost that were not degraded by autoclaving may have functioned as defensive substances against pathogenic fungi. These substances may contribute to the suppressiveness of FYM soil on plates at low dilutions by the co-cultivation method. Toyota and Kimura (14) found that bacterial strains attached to the chlamydospores of *F. oxysporum* f. sp. *raphani* and lysed them, but did not inhibit mycelial growth. In this case, a biotic factor was responsible for suppressiveness; however, it was not possible to evaluate suppressiveness using the co-cultivation method.

The co-cultivation method has some limitations, *i.e.* it cannot be applied when the cause of suppressiveness is abiotic. In addition, the degree of suppression achieved with the combination of a pathogenic strain of *F. oxysporum* and a crop cannot be extrapolated to other combinations of diseases and crops; the pathogenic strain of *F. oxysporum* responsible for each combination of disease and crop needs to be used. However, a merit is that a quick and easy estimation of soil suppressiveness is possible by using common equipment only such as an autoclave and a clean bench within 10 d. Therefore, the co-cultivation method may be useful for predicting and diagnosing suppressiveness against soil-borne diseases.

## Conclusions

We applied the co-cultivation method of *F. oxysporum* and soil microorganisms to soil that exhibited suppressiveness against soil-borne diseases due to the long-term application of organic amendments. A correlation was found between the disease incidence of spinach and growth degree of *F. oxysporum* f. sp. *spinaciae*, indicating that it is possible to evaluate suppressiveness against spinach wilt disease by the co-cultivation method. However, there were some exceptions where soil showed a low disease incidence, but the growth of *F. oxysporum* was not suppressed on agar plates, suggesting the limitations of this method in its applicability. Further investigations are needed to elucidate the relationship between suppressiveness and the biological and chemical properties of soil in addition to the growth degree of *F. oxysporum* in order to comprehensively predict and diagnose suppressiveness.

## Supplementary Material
