# Supplementary material for: Evaluation of Suppressiveness of Soils Exhibiting Soil-Borne Disease Suppression after Long-Term Application of Organic Amendments by the Co-cultivation Method of Pathogenic Fusarium oxysporum and Indigenous Soil Microorganisms
Source: Microbes Environ. 2018 Mar 29;33(1):58–65. doi: 10.1264/jsme2.ME17072 (PMC5877344; doi:10.1264/jsme2.ME17072)
Supplement: Supplementary file 1 [file 33_58_s1.pdf]

Supporting information

Evaluation of Suppressiveness of Soils Exhibiting Soil-Borne Disease Suppression after Long-Term Application of Organic Amendments by the Co-cultivation Method of Pathogenic *Fusarium oxysporum* and Indigenous Soil Microorganisms

Masahiro Mitsuboshi, Yuuzou Kioka, Katsunori Noguchi, and Susumu Asakawa

Materials and Methods

Cultivation and fertilization of the field

The field at Aichi prefecture had two croppings in a year. From 1987 to 1989, sweet corn (*Zea mays* L. convar. *saccharata*), radish (*Raphanus sativus* L. var. *longipinnatus*), and "Kyoumizuna" (*Brassica rapa* L. var. *laciniifolia*) + "Hinona" (*Brassica rapa* L. var. *akana*) were grown and from 1990 to 1997 melon (*Cucumis melo* L.) in spring and cabbage (*Brassica oleracea* L. var. *capitata*) in autumn were grown (1). Since 1998 sweet corn in spring and Chinese cabbage (*Brassica rapa* L. var. *pekinensis*) in autumn have been cultivated.

The field at Ibaraki prefecture had one or two croppings in a year. From 1987 to 1996, "Edamame" (soy bean, *Glycine max* [L.] Merr.) (N-P_2O_5-K_2O = 75-120-100 kg ha^{-1}) in spring and Chinese cabbage (N-P_2O_5-K_2O = 200-250-200 kg ha^{-1}) in autumn were cultivated and from 1997 to 2002 tomato (*Solanum lycopersicum* Mill.) (N-P_2O_5-K_2O = 100 to 200-100 to 250-100 to 200 kg ha^{-1}) was mono cropped. From 2003 to 2012, bok-choy (*Brassica rapa* L. var. *chinensis*) (N-P_2O_5-K_2O = 100-100-100 kg ha^{-1}), carrot (*Daucus carota* L. subsp. *sativus*) (N-P_2O_5-K_2O = 60-150-60 kg ha^{-1}, "Komatsuna" (*Brassica rapa* L. var.

perviridis) (N-P_2O_5-K_2O = 150-150-150 kg ha^{-1}), radish (N-P_2O_5-K_2O = 80-150-80 kg ha^{-1}), "Edamame" (N-P_2O_5-K_2O = 50-100-100 kg ha^{-1}), spinach (*Spinacia oleracea* L.) (N-P_2O_5-K_2O = 150-150-150 kg ha^{-1}), corn (*Zea mays*) (N-P_2O_5-K_2O = 100-100-100 kg ha^{-1}), Chinese cabbage (N-P_2O_5-K_2O = 200-250-200 kg ha^{-1}), potato (*Solanum tuberosum* L.) (N-P_2O_5-K_2O = 100-200-100 kg ha^{-1}, and onion (*Allium cepa* L.) (N-P_2O_5-K_2O = 150-250-150 kg ha^{-1}) were cultivated and sorghum (*Sorghum bicolor* [L.] Moench) was cultivated without fertilization in autumn of 2008. "Edamame" (N-P_2O_5-K_2O = 80-100-150 kg ha^{-1}) in spring of 2013, cabbage (N-P_2O_5-K_2O = 90-150-90 kg ha^{-1}) in spring of 2014, and Chinese cabbage (N-P_2O_5-K_2O = 250-250-250 kg ha^{-1}) in autumn of 2014 were grown. Application amount of organic fertilizer for each cultivation in Ibaraki soil is shown in Table S3. Until the spring of 1996, the amount of applied original fertilizer was adjusted to account for 2/3 of nitrogen in basal fertilizer and the rest 1/3 of nitrogen was supplemented with ammonium sulfate. Since the autumn of 1996, the amount of applied organic fertilizer has been adjusted to the amount of nitrogen in the basal fertilizer. Shortage amounts of phosphorus and potassium in the organic fertilizers were supplemented with compound inorganic fertilizers (calcium superphosphate and potassium chloride), while the excess amounts of phosphorus and potassium were stood when the amounts were over the required amounts mentioned above. Top dressing was applied with compound inorganic fertilizers (ammonium sulfate and potassium chloride) when it was needed.

References

1 Katayama, A., H. Y. Hu, M. Nozawa, H. Yamakawa, and K. Fujie. 1998. Long-term changes in

microbial community structure in soils subjected to different fertilizing practices revealed by quinone profile analysis. Soil. Sci. Plant Nutr. 44:559–569.

Table S1. Chemical and microbiological characteristics of soils used in the experiment[a]

	Aichi soil					Ibaraki soil									
	NF	CF	CF+ FYM	CRC	FYM	Cont		RSM		FM		SBM		Mix	
Chemical characteristics															
pH (H_2O)	6.59	6.15	6.53	5.83	6.55	5.26	±0.11	6.30	±0.11**	6.69	±0.08**	6.93	±0.04**	6.85	±0.03**
EC (dS m^{-1})	0.03	0.16	0.13	0.03	0.03	0.20	±0.01	0.16	±0.00**	0.10	±0.00**	0.10	±0.01**	0.10	±0.01**
NH_3-N (mg kg^{-1})	5.1	5.1	4.1	7.2	5.2	7.8	±1.25	9.2	±1.54	8.5	±1.58	8.2	±0.93	8.9	±1.71
NO_3-N (mg kg^{-1})	2.0	5.1	13.2	31.7	38.6	9.6	±1.64	8.2	±1.80	12.4	±5.96	10.3	±2.28	15.4	±1.88
Available P (P_2O_5 mg kg^{-1}) [b]	60	162	1,290	153	5,730	50	±14.3	64	±16.5	110	±24.1	1,570	±192**	526	±138
Exchangeable K (K_2O mg kg^{-1})	102	168	724	328	2,560	435	±21.8	664	±21.2**	707	±20.6**	588	±49.3*	722	±39.4**
Exchangeable Ca (CaO mg kg^{-1})	852	864	2,030	1,230	5,510	1,440	±55.5	2,800	±134**	3,110	±67.7**	3,810	±10.2**	3,910	±76.1**
Exchangeable Mg (MgO mg kg^{-1})	73	224	444	353	1,550	109	±17.1	459	±13.0*	430	±20.2*	654	±13.9**	559	±19.3**
Phosphate absorption coefficient (P_2O_5 mg kg^{-1})	6,960	7,760	7,080	7,520	11,600	28,100	±6,740	25,900	±5,650*	25,400	±2,160*	24,800	±5,180**	24,600	±5,420**
CEC ($cmol_c$ kg^{-1})	5.2	5.8	10.3	10.7	25.3	17.5	±10.0	19.9	±6.04	19.4	±13.7	20.9	±9.25	21.5	±7.67
Microbial characteristics															
Bacteria (×10^4 cfu g^{-1})	760	400	3100	3300	3500	1,900	±751	2,600	±153	4,130	±481	3,500	±656	2,830	±546
Actinomycetes (×10^4 cfu g^{-1})	340	290	480	3600	2400	280	±80.8	1,660	±626*	1,970	±33.3*	3,870	±240**	1,830	±133*
Fungi (×10^3 cfu g^{-1})	51	73	68	1600	45	64	±8.41	157	±71.3	98	±16.8	96	±27.2	68	±5.70

[a] pH, EC and microbial characteristics were measured in moist soil and the other characteristics were measured in air-dried soil.

[b] Truog method.

NF, unfertilized; CF, chemical fertilizer; CF+FYM, chemical fertilizer and 40 t ha^{-1} y^{-1} farmyard manure; CRC, chemical fertilizer and 40 t ha^{-1} y^{-1} coffee residue compost; FYM, 400 t ha^{-1} y^{-1} farmyard manure. Cont, compound inorganic fertilizers; RSM, 940-4,700 kg ha^{-1} rapeseed meal; FM, 710-3,600 kg ha^{-1} fish meal; SBM, 1,300-6,300 kg ha^{-1} steamed bone meal; Mix, 930-4,600 kg ha^{-1} mixture of rapeseed meal, fish meal, and steamed bone meal. * and ** indicate significant differences from Cont at $P<0.05$ and $P<0.01$; Dunnett's test, respectively. Data in Ibaraki soil are shown with SE (n = 3).

Table S2. Statistics and diversity indexes of bacterial community in the soil used in the experiment (Ibaraki soil)

	Cont[a]			RSM[a]			FM[a]			SBM[a]			Mix[b]
Statistics													
Observed species	1,060	±	29	1,290	±	25	1,250	±	24	1,220	±	34	1,250
Singles	609	±	25	771	±	27	759	±	23	716	±	26	704
Diversity indexes													
Chao1	2,260	±	60	2,920	±	191	2,930	±	113	2,600	±	85	2,440
ACE	2,350	±	133	2,980	±	137	3,030	±	93	2,800	±	96	2,660
Shannon index	8.42	±	0.03	8.85	±	0.05	8.81	±	0.03	8.85	±	0.06	8.89
Simpson index	0.99	±	0.00	0.99	±	0.00	0.99	±	0.00	0.99	±	0.00	0.99

[a]Data are shown with SE (n = 3). There was no significant difference from Cont.
[b]Measurement was conducted for one of the triplicate plots.
Cont, compound inorganic fertilizers; RSM, 940-4,700 kg ha^{-1} rapeseed meal; FM, 710-3,600 kg ha^{-1} fish meal; SBM, 1,300-6,300 kg ha^{-1} steamed bone meal; Mix, 930-4,600 kg ha^{-1} mixture of rapeseed meal, fish meal, and steamed bone meal.

Table S3 Application amount of organic fertilizer for each cultivation in Ibaraki soil

			**RSM**	**FM**	**SBM**	**Mix**
				(kg ha^{-1})		
**1987-1996**[a]	spring	Edamame	940	710	1,300	930
	autumn	Chinese cabbage	2,500	1,900	3,300	2,500
**1997-2002**	spring	tomato	1,900-3,800	1,400-2,900	2,500-5,000	1,900-3,700
**2003**	spring	bok-choy	1,900	1,400	2,500	1,900
	autumn	carrot	1,100	860	1,500	1,100
**2004**	spring	Komatsuna	2,800	2,100	3,800	2,800
	autumn	radish	1,500	1,100	2,000	1,500
**2005**	spring	Edamame	940	710	1,300	930
**2006**	autumn	spinach	2,800	2,100	3,800	2,800
**2007**	spring	edamame	940	710	1,300	930
	autumn	Chinese cabbage	2,500	1,900	3,300	2,500
**2008**	spring	Edamame	940	710	1,300	930
	autumn	sorghum[b]	-	-	-	-
**2009**	spring	corn	1,900	1,400	2,500	1,900
	autumn	Chinese cabbage	2,500	1,900	3,300	2,500
**2010**	spring	Edamame	940	710	1,300	930
	autumn	onion	2,800	2,100	3,800	2,800
**2011**	spring	potato	1,900	1,400	2,500	1,900
	autumn	carrot	1,100	860	1,500	1,100
**2012**	spring	Edamame	940	710	1,300	930
	autumn	radish	1,500	1,100	2,000	1,500
**2013**	spring	Edamame	1,500	1,100	2,000	1,500
	autumn	carrot	1,100	860	1,500	1,100
**2014**	spring	cabbage	1,700	1,300	2,300	1,700
	autumn	Chinese cabbage	4,700	3,600	6,300	4,600

[a] The amount of applied organic fertilizer was adjusted to account for 2/3 of nitrogen in basal fertilizer.
[b] Sorghum was cultivated without fertilization in autumn of 2008.

Figure legends

Fig. S1. Principal component analysis (PCA) performed on chemical and microbiological characteristics. pH, EC, NH_3-N, exchangeable Mg, bacterial density, and fungal density were used for the analysis. NF, unfertilized; CF, chemical fertilizer; CF+FYM, chemical fertilizer and 40 t ha^{-1} y^{-1} farmyard manure; CRC, chemical fertilizer and 40 t ha^{-1} y^{-1} coffee residue compost; FYM, 400 t ha^{-1} y^{-1} farmyard manure. Cont, compound inorganic fertilizers; RSM, 940-4,700 kg ha^{-1} rapeseed meal; FM, 710-3,600 kg ha^{-1} fish meal; SBM, 1,300-6,300 kg ha^{-1} steamed bone meal; Mix, 930-4,600 kg ha^{-1} mixture of rapeseed meal, fish meal, and steamed bone meal.

Fig. S2. Growth of spinach (A, C) and Boston lettuce (B, D) in Aichi soil (A, B) and Ibaraki soil (C, D). NF, unfertilized; CF, chemical fertilizer; CF+FYM, chemical fertilizer and 40 t ha^{-1} y^{-1} farmyard manure; CRC, chemical fertilizer and 40 t ha^{-1} y^{-1} coffee residue compost; FYM, 400 t ha^{-1} y^{-1} farmyard manure. Cont, compound inorganic fertilizers; RSM, 940-4,700 kg ha^{-1} rapeseed meal; FM, 710-3,600 kg ha^{-1} fish meal; SBM, 1,300-6,300 kg ha^{-1} steamed bone meal; Mix, 930-4,600 kg ha^{-1} mixture of rapeseed meal, fish meal, and steamed bone meal.

Fig. S3. Influence of sterilization of soil by autoclaving on disease incidence in the inoculation experiment. Disease incidence of spinach by *Fusarium oxysporum* f. sp. *spinaciae* and Boston lettuce by *F. oxysporum* f. sp. *lactucae*. RSM, 940-4,700 kg ha^{-1} rapeseed meal; CRC, chemical fertilizer and 40 t ha^{-1} y^{-1} coffee

residue compost; FYM, 400 t ha^{-1} y^{-1} farmyard manure; S indicates sterilized soil. Values show the median with the upper and lower quartile points (n = 9). * and ** indicate significant differences at $P<0.05$ and $P<0.01$ (Steel's test). ns indicates not significant (Steel's test).

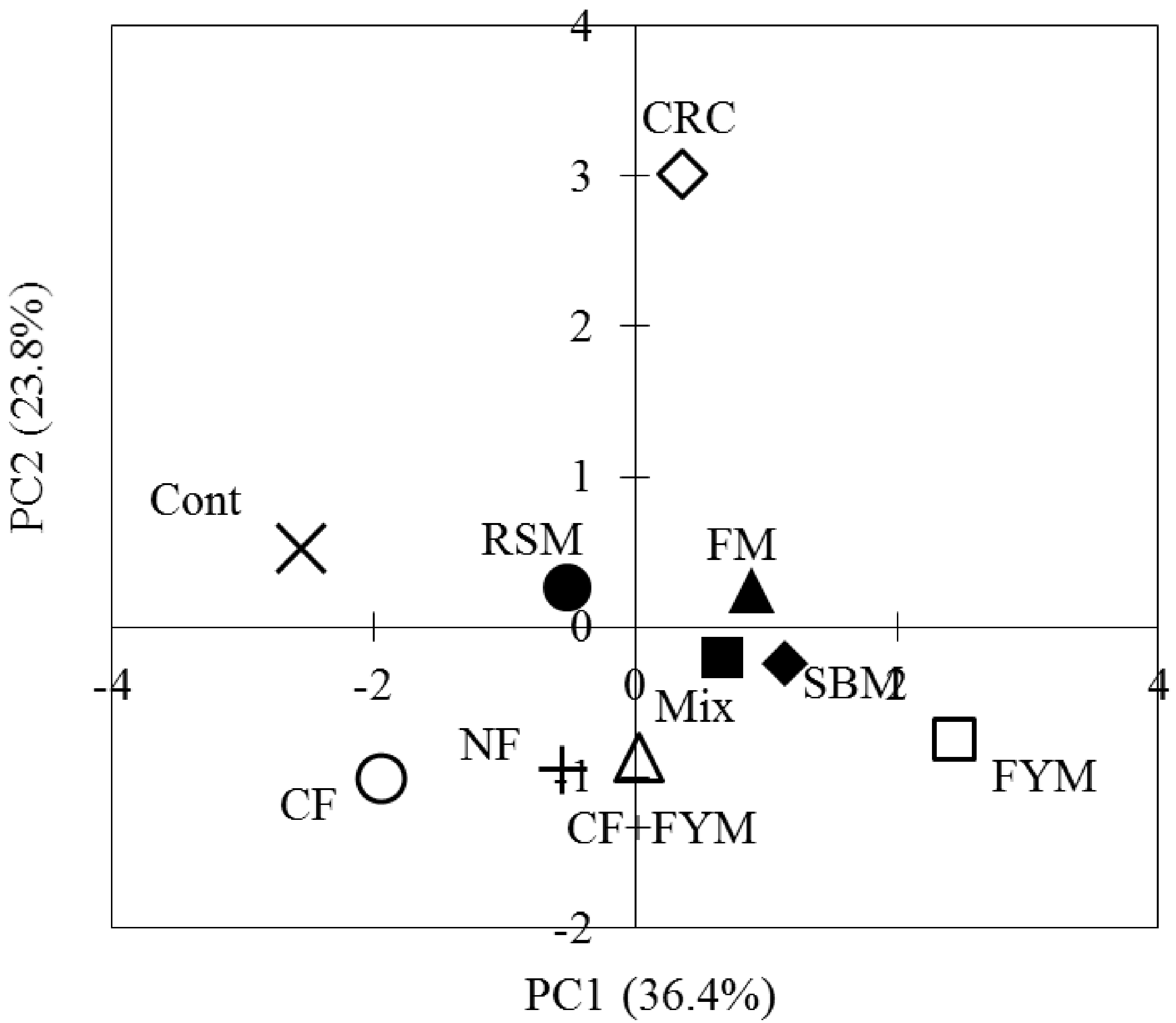

Fig. S1 Mitsuboshi

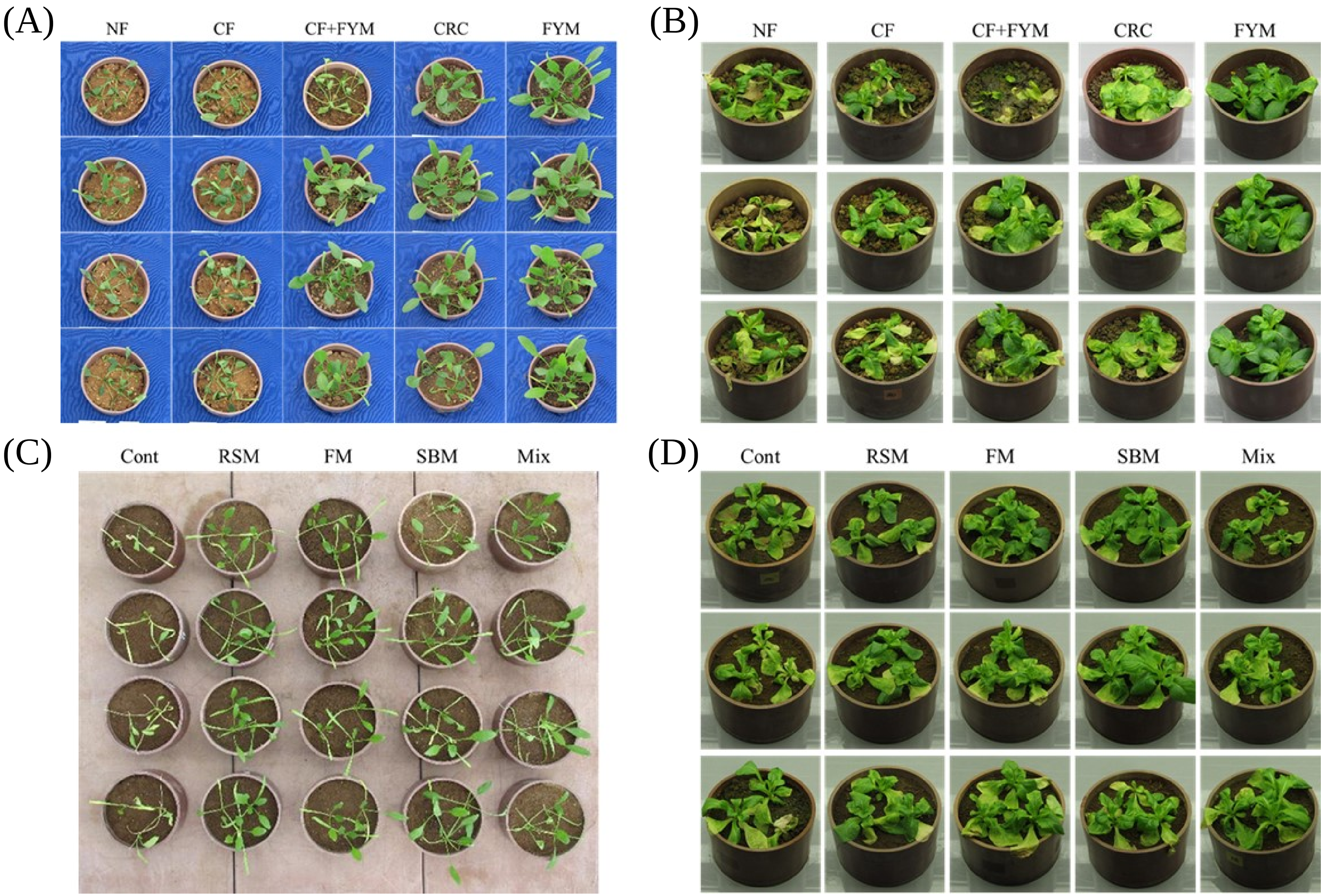

Fig. S2 Mitsuboshi

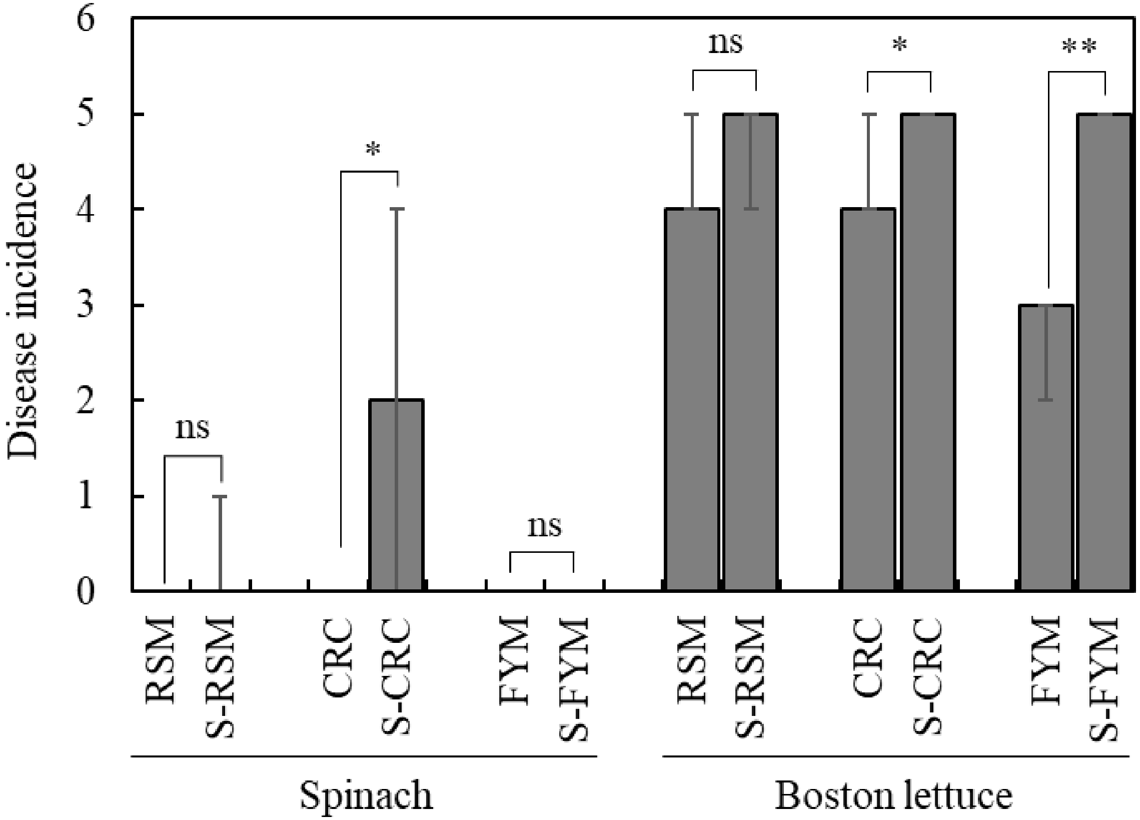

Fig. S3 Mitsuboshi
